# Noncollapsibility and its role in quantifying confounding bias in logistic regression

**DOI:** 10.1186/s12874-021-01316-8

**Published:** 2021-07-05

**Authors:** Noah A. Schuster, Jos W. R. Twisk, Gerben ter Riet, Martijn W. Heymans, Judith J. M. Rijnhart

**Affiliations:** 1 Department of Epidemiology and Data Science, Amsterdam Public Health Research Institute, Amsterdam UMC - Location VU University Medical Center, De Boelelaan 1117, Amsterdam, The Netherlands; 2 Center of Expertise Urban Vitality, Faculty of Health, Amsterdam University of Applied Sciences, Amsterdam, The Netherlands; 3 Department of Cardiology, Amsterdam Public Health Research Institute, Amsterdam UMC - Location AMC, Meibergdreef 9, Amsterdam, The Netherlands

**Keywords:** Logistic regression, Confounding, Noncollapsibility, Confounder-adjustment, Univariable regression analysis, Multivariable regression analysis, Inverse probability weighting, Conditional effect, Marginal effect

## Abstract

**Background:**

Confounding bias is a common concern in epidemiological research. Its presence is often determined by comparing exposure effects between univariable- and multivariable regression models, using an arbitrary threshold of a 10% difference to indicate confounding bias. However, many clinical researchers are not aware that the use of this change-in-estimate criterion may lead to wrong conclusions when applied to logistic regression coefficients. This is due to a statistical phenomenon called noncollapsibility, which manifests itself in logistic regression models. This paper aims to clarify the role of noncollapsibility in logistic regression and to provide guidance in determining the presence of confounding bias.

**Methods:**

A Monte Carlo simulation study was designed to uncover patterns of confounding bias and noncollapsibility effects in logistic regression. An empirical data example was used to illustrate the inability of the change-in-estimate criterion to distinguish confounding bias from noncollapsibility effects.

**Results:**

The simulation study showed that, depending on the sign and magnitude of the confounding bias and the noncollapsibility effect, the difference between the effect estimates from univariable- and multivariable regression models may underestimate or overestimate the magnitude of the confounding bias. Because of the noncollapsibility effect, multivariable regression analysis and inverse probability weighting provided different but valid estimates of the confounder-adjusted exposure effect. In our data example, confounding bias was underestimated by the change in estimate due to the presence of a noncollapsibility effect.

**Conclusion:**

In logistic regression, the difference between the univariable- and multivariable effect estimate might not only reflect confounding bias but also a noncollapsibility effect. Ideally, the set of confounders is determined at the study design phase and based on subject matter knowledge. To quantify confounding bias, one could compare the unadjusted exposure effect estimate and the estimate from an inverse probability weighted model.

**Supplementary Information:**

The online version contains supplementary material available at 10.1186/s12874-021-01316-8.

## Background

In observational studies, the exposure levels are often influenced by characteristics of the study subjects. As a result, differences in background characteristics between exposed and unexposed individuals may exist. If these characteristics are also associated with the outcome, crude comparison of the average outcomes in both exposure groups does not yield an unbiased estimate of the exposure effect [1–5]. Therefore, to obtain unbiased effects, adjustment for this imbalance in background characteristics is necessary. This is also called adjustment for confounding.

When selecting confounders for adjustment, researchers often use statistical methods to quantify the confounding bias. That is, oftentimes the confounding bias is quantified by comparing the exposure effect between a univariable- and a multivariable regression model, also called the change-in-estimate criterion [4, 6, 7]. However, this method may lead to wrong conclusions about the presence and magnitude of confounding bias, as in logistic regression covariates may affect the effect estimate through two separate mechanisms: through confounding when covariates are associated with both the exposure and the outcome, and through noncollapsibility which is present when covariates are associated with the outcome [8]. The total difference between the effect estimate from a univariable- and multivariable regression model may therefore be decomposed into an estimate of confounding bias and an estimate of the noncollapsibility effect [7, 9]. Furthermore, even in the absence of confounding the exposure effect coefficients from both models might still differ. Thus, the change-in-estimate may misrepresent the true confounding bias [4].

Various rescaling methods have been proposed in the social sciences literature, which aim to equalize the scales of the effect estimates from a univariable and a multivariable regression model [10–13]. However, when applied to effect estimates from a logistic regression, these rescaling measures are approximate rather than exact [10, 11, 14]. Janes et al. [9] and Pang et al. [7] proposed an exact measure of confounding bias for logistic regression models. This measure is based on the comparison of the effect estimates from a univariable regression model and an inverse probability weighted (IPW) model. The latter is another popular method to adjust for confounding.

Noncollapsibility may not only affect the differences between the effect estimates from a univariable- and multivariable regression model, it also causes differences between the effect estimates from a multivariable regression model and an IPW model. Whereas multivariable regression and IPW provide the same effect estimates in linear regression, this does not necessarily hold for logistic regression [7, 9, 15]. That is, when a noncollapsibility effect is present, multivariable regression adjustment and IPW both yield valid estimates of the confounder-adjusted exposure effect, but their magnitude and interpretation differ [7, 16, 17]. Therefore, the difference between the effect estimates from a multivariable regression model and IPW can be used to quantify the magnitude of noncollapsibility.

Because noncollapsibility is a relatively unknown mechanism among clinical researchers, many are unaware that the change-in-estimate criterion may lead to wrong conclusions about the presence and magnitude of confounding bias. Therefore, this paper aims to clarify the role of noncollapsibility in logistic regression and to provide guidance in determining the presence of confounding bias. First, we review the different confounder-adjustment methods and provide a detailed explanation of the noncollapsibility effect. Then, we use a Monte Carlo simulation study to uncover patterns of confounding bias and noncollapsibility effects in logistic regression. Subsequently, using an empirical data example, we demonstrate that the change-in-estimate criterion to determine confounding bias may be misleading. Finally, we provide guidance in determining the set of confounders and quantifying confounding bias.

## Confounder adjustment and noncollapsibility

The presence and magnitude of confounding bias for models with a binary outcome is commonly determined by comparing the exposure effect estimates from a univariable- (Eq. 1) and multivariable (Eq. 2) logistic regression model:

1 $$logit\left(Pr\left(Y=1|X\right)\right)= {i}_{1}+{\beta }_{1}X$$

2 $$logit\left(Pr\left(Y=1|X,{C}_{1},\dots ,{C}_{n}\right)\right)= {i}_{2}+ {\beta }_{1}^{^{\prime}}X+{{\beta }_{2}^{^{\prime}}C}_{1}+\cdots + {\beta }_{n+1}^{^{\prime}}{C}_{n}$$

where in both equations, $Y$ and $X$ represent the outcome and exposure variables and ${i}_{1}$ and ${i}_{2}$ represent the intercept terms, respectively. In Eq. 1, ${\beta }_{1}$ represents the *unadjusted* exposure effect estimate. In Eq. 2, ${\beta }_{1}^{^{\prime}}$ represents the multivariable confounder-adjusted exposure effect estimate and ${\beta }_{2}^{^{\prime}}$ to ${\beta }_{n+1}^{^{\prime}}$ are the coefficients corresponding to observed background covariates ${C}_{1}$ to ${C}_{n}$. When ${C}_{1}$ to ${C}_{n}$ are truly confounders, then ${\beta }_{1}$ will be a biased estimate of the causal exposure-outcome effect. Assuming that Eq. (2) contains all confounders of the exposure-outcome effect, ${\beta }_{1}^{^{\prime}}$ will have a causal interpretation. In practice researchers often determine the magnitude of confounding as the change in estimate, which is computed as the difference between ${\beta }_{1}^{^{\prime}}$ and ${\beta }_{1}$.When using the change-in-estimate criterion to determine the presence of confounding bias typically a 10% difference between ${\beta }_{1}^{^{\prime}}$ and ${\beta }_{1}$ is used in practice as an arbitrary threshold indicating confounding due to covariates ${C}_{1}$ to ${C}_{n}$ in the association between $X$ and $Y$ [6, 18, 19].

When based on logistic regression, ${\beta }_{1}^{^{\prime}}-{\beta }_{1}$ may not only represent confounding bias but also a noncollapsibility effect. This noncollapsibility effect is sometimes also referred to as a form of the Simpson’s paradox [16]. The noncollapsibility effect is caused by a difference in the scale on which ${\beta }_{1}$ and ${\beta }_{1}^{^{\prime}}$ are estimated. In linear regression, the total variance is the same for nested models: when the explained variance increases through adding a covariate to the model, the *un*explained variance decreases by the same amount. As a result, effect estimates from nested linear models are on the same scale and thus *collapsible.* In logistic regression, however, the unexplained variance has a fixed value of 3.29 [8]. Adding covariates that are associated with the outcome (e.g., confounders) increases the explained variance and forces the total variance of *Y* to increase. When the total variance of *Y* increases, the scale of the estimated coefficients changes, causing negative exposure effects to become more negative and positive exposure effects more positive. This change in scales is called the *noncollapsibility* effect [5, 7, 8]. Thus, to determine confounding bias, exposure effect estimates cannot be simply compared between nested logistic regression models as the difference might not only reflect confounding bias but also a noncollapsibility effect [8]. The noncollapsibility effect also occurs when a covariate is associated with outcome *Y* but not with exposure *X* (i.e., when the covariate is not a confounder). The change in estimate then represents the noncollapsibility effect only, falsely indicating the presence of confounding bias. To preserve space in the main text, a hypothetical example illustrating how the change in estimate might be affected by the noncollapsibility effect in the absence of confounding is given in additional file A. An explanation of noncollapsibility based on a contingency table is provided by for example Pang et al. [7].

Recent studies by Janes et al. and Pang et al. presented an exact estimate of confounding bias unaffected by noncollapsibility based on logistic regression [7, 9], using the difference between the univariable exposure effect estimate and the effect estimate from an IPW model. With IPW, confounding bias is eliminated by creating a pseudo-population in which each covariate combination is balanced between both exposure groups [20–22]. When there is perfect covariate balance there is no longer an association between covariates ${C}_{1}$ to ${C}_{n}$ and exposure status $X$. This pseudo-population can be created by weighting subjects so that for each combination of baseline covariates the sums of contributions for both exposure groups are equal [1, 20]. These weights are the inverse of the probability that a subject was exposed, i.e. the inverse of a propensity score [23].

The propensity score is the predicted probability of endorsing the exposure, which can be estimated using Eq. 3:

3 $$PS=Pr\left(X=1|C\right)= \frac{1}{1+{e}^{-\left({i}_{3}+{\lambda }_{1}{C}_{1}+\dots +{\lambda }_{n}{C}_{n}\right)}}$$

where *X* represents exposure, ${i}_{3}$ is the model intercept and ${\lambda }_{1}$ to ${\lambda }_{n}$ are regression coefficients corresponding to covariates ${C}_{1}$ to ${C}_{n}$. The propensity score methodology can also be extended to continuous exposure variables using the Generalized Propensity Score (GPS), which has a similar balancing property to the classic propensity score. For more information on how to perform propensity score analysis with a continuous exposure variable, see Hirano (2004) and Imai (2004) [24, 25].

For exposed subjects, the weight is calculated as $\frac{1}{PS}$ and for unexposed subjects as $\frac{1}{1-PS}$ [1, 20, 22]. Using these calculations, subjects with a propensity score close to 0 end up with large weights, and subjects with a propensity score close to 1 end up with small weights. Because in some situations these weights cause the IPW model to be unstable, stabilized weights have been proposed [26]. For exposed subjects, the stabilized weight is calculated as $\frac{p}{PS}$ and for unexposed subjects as $\frac{1- p}{1-PS}$, where *p* is the probability of exposure without considering covariates ${C}_{1}$ to ${C}_{n}$ [2, 26]. Subsequently, a weighted regression analysis with exposure *X* as the only independent variable is carried out. We call the confounder-adjusted exposure effect estimate from the IPW model ${\beta }_{1}^{*}$.

### Difference between IPW- and multivariable confounder-adjusted exposure effect estimates

Multivariable regression adjustment and IPW provide identical exposure effect estimates when based on linear regression, but not when based on logistic regression [15, 27]. The difference between the IPW confounder-adjusted exposure effect estimate ${\beta }_{1}^{*}$ and the multivariable confounder-adjusted exposure effect estimate ${\beta }_{1}^{^{\prime}}$ is caused by noncollapsibility, and the difference between the unadjusted exposure effect estimate ${\beta }_{1}$ and ${\beta }_{1}^{*}$ provides a measure of confounding bias [7, 9, 14]. This is because in an IPW model the total variance remains equal to the total variance of the unadjusted model, while in a multivariable regression model the addition of variables to the model leads to higher variance, changing the scale of the exposure effect estimate. This means that when there is confounding in a logistic regression model, multivariable regression analysis and IPW lead to different confounder-adjusted estimates of the exposure effect. Although ${\beta }_{1}^{^{\prime}}$ and ${\beta }_{1}^{*}$ are both valid estimates, they apply to different target populations and have their own respective interpretation [8, 27].

## Simulation study

### Simulation methods

A Monte Carlo simulation study was designed to investigate patterns of confounding bias and noncollapsibility effects in logistic regression. The R programming language version 4.0.2 [28] and STATA statistical software release 14 [29] were used to generate and analyze the data, respectively.

Three continuous covariates were generated from a standard normal distribution. The dichotomous exposure and outcome were generated from a binomial distribution conditional on the covariates and the covariates and exposure, respectively. Sample sizes were 250, 500, 750 and 1000. The parameter values for the exposure-outcome effect, confounder-exposure effect and the confounder-outcome effect were set to -1.42, -0.92, -0.38, 0, 0.38, 0.92 and 1.42. This way, the conditions reflected situations with combinations of zero effects, and positive and negative small (-0.38 and 0.38), medium (-0.92 and 0.92) and large (-1.42 and 1.42) effect sizes were mimicked [30]. The total number of conditions was 1,372 with 1,000 repetitions per condition, resulting in 1,372,000 observations. Subsequently, we estimated the unadjusted exposure effect estimate ${\beta }_{1}$, the multivariable confounder-adjusted exposure effect estimate ${\beta }_{1}^{^{\prime}}$ and the IPW confounder-adjusted exposure effect estimate ${\beta }_{1}^{*}$ based on the simulated data. From these effect estimates we computed the change in estimate, the confounding bias and the noncollapsibility effect. The simulation code is available in additional file B.

### Simulation scenarios

**Fig. 1:**
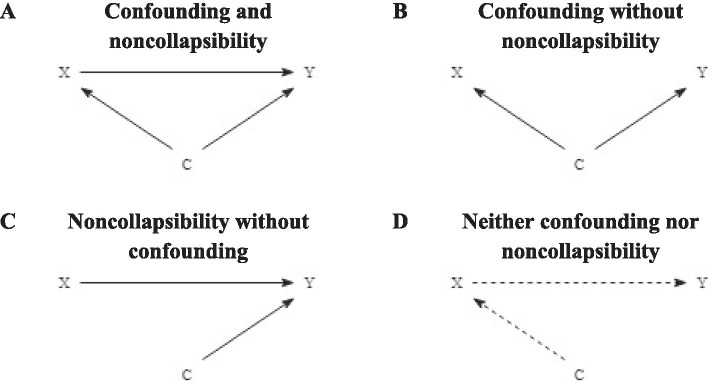
Directed acyclic graphs of the four possible scenarios into which each simulated condition can be classified. Panel **A**: both confounding and noncollapsibility. Panel **B**: confounding without noncollapsibility. Panel **C**: noncollapsibility without confounding. Panel **D**: neither confounding nor noncollapsibility. *C* represents three continuous covariates, *X* represents the dichotomous exposure and *Y* represents the dichotomous outcome. The dotted line in panel D between the covariates and the exposure and between the exposure and the outcome indicate there may or may not be an association

We expected to observe four scenarios based on the simulated data. In the first scenario (Fig. 1a), the covariates are associated with both the exposure and the outcome. In this scenario there will be both confounding bias (${\beta }_{1}$ – ${\beta }_{1}^{*}$) and a noncollapsibility effect (${\beta }_{1}^{*}$ – ${\beta }_{1}^{^{\prime}}$). Because the exposure-outcome effect is simulated to be positive and negative, we also expect to see positive and negative noncollapsibility effect estimates. This means that ${\beta }_{1}^{^{\prime}}-{\beta }_{1}$ might result in an under- or overestimation of the true confounding effect [8]. In the second scenario (Fig. 1b) the covariates are associated with both the exposure and outcome, but exposure and outcome are not associated with each other. In this scenario, any differences between ${\beta }_{1}^{^{\prime}}$ and ${\beta }_{1}$ are fully explained by the covariates, so there is confounding bias without a noncollapsibility effect [8, 15]. In the third scenario (Fig. 1c), the covariates are only associated with the outcome. In this scenario there is a noncollapsibility effect but no confounding bias. In real-life situations with this structure, using the change-in-estimate criterion may lead one to conclude that the covariates are confounders in the relation between the exposure and the outcome although the difference between ${\beta }_{1}^{^{\prime}}$ and ${\beta }_{1}$ is caused entirely by the noncollapsibility effect [7, 8]. In the fourth scenario (Fig. 1d), the covariates may be associated with the exposure, but not with the outcome. In this scenario, there is neither confounding bias nor a noncollapsibility effect and ${\beta }_{1}$, ${\beta }_{1}^{^{\prime}}$ and ${\beta }_{1}^{*}$ are identical. This scenario is also called *strict* collapsibility [15, 31, 32].

### Simulation results

The difference between ${\beta }_{1}^{^{\prime}}$ and ${\beta }_{1}$ can be negative, zero or positive, depending on the magnitude of the confounding bias and the noncollapsibility effect (Table 1). Only when there was no noncollapsibility effect (i.e., ${\beta }_{1}^{*}-$ ${\beta }_{1}^{^{\prime}}=0$), the change in estimate equaled the estimate of confounding bias. The noncollapsibility effect was zero when the exposure-outcome effect was zero and the confounder-exposure and confounder-outcome effects were both non-zero. When the exposure-outcome effect was also non-zero, the difference between ${\beta }_{1}^{^{\prime}}$ and ${\beta }_{1}$ reflected both confounding bias and the noncollapsibility effect. In those situations, the change-in-estimate criterion could both under- and overestimate the true confounding bias. When the confounding bias and noncollapsibility effect had similar signs, i.e. both were positive or negative, ${\beta }_{1}^{^{\prime}}-{\beta }_{1}$ overestimated the true confounding bias. When the confounding bias and noncollapsibility effect had opposites signs, i.e. one was positive while the other was negative, the true confounding bias could be under- or overestimated by ${\beta }_{1}^{^{\prime}}-{\beta }_{1}$, depending on the magnitude of the confounding bias and noncollapsibility effect. Thus, when the exposure-outcome effect is non-zero, the change-in-estimate criterion might falsely indicate the presence of confounding or it might under- or overestimate the true confounding bias. Patterns of confounding bias and the noncollapsibility effect were similar across sample sizes and will be described below.

**Table 1:** Difference between univariable- and multivariable exposure effects as combination of confounding bias and the noncollapsibility effect

Difference between multivariable- and univariable effect estimate $({{\varvec{\beta}}}_{1}^{\boldsymbol{^{\prime}}}-{{\varvec{\beta}}}_{1}$)	Confounding bias (${{\varvec{\beta}}}_{1}-{{\varvec{\beta}}}_{1}^{\boldsymbol{*}}$)	Noncollapsibility effect (${{\varvec{\beta}}}_{1}^{\boldsymbol{*}}-{{\varvec{\beta}}}_{1}^{\boldsymbol{^{\prime}}}$)
Negative	Negative value	Negative value
	Zero	Negative value
	Negative value	Zero
	Positive value	Greater negative value than the positive confounding bias value
	Greater negative value than the positive noncollapsibility effect value	Positive value
Zero	Zero	Zero
	Equal positive value as the negative noncollapsibility effect value	Equal negative value as the positive confounding bias value
	Equal negative value as the positive noncollapsibility effect value	Equal positive value as the negative confounding bias value
Positive	Positive value	Positive value
	Zero	Positive value
	Positive value	Zero
	Negative value	Greater positive value than the negative confounding bias value
	Greater positive value than the negative noncollapsibility effect value	Negative value

### Confounding bias

**Fig. 2:**
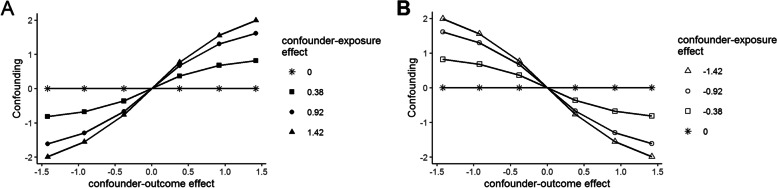
True confounding bias (${{\varvec{\beta}}}_{1}-{{\varvec{\beta}}}_{1}^{\boldsymbol{*}}$) as a function of the confounder-outcome effect collapsed over all sample sizes. Panel **A**: each line represents a positive confounder-exposure effect. Panel **B**: each line represents a negative confounder-exposure effect

Figure 2 plots confounding bias (${\beta }_{1}-{\beta }_{1}^{*}$) as a function of the confounder-outcome effect with the lines in panel A representing positive confounder-outcome effects of various magnitudes and the lines in panel B representing negative confounder-outcome effects of various magnitudes. Confounding bias was positive when the confounder-exposure effect and the confounder-outcome effect were both positive (panel A, first quadrant) and when they were both negative (panel B, second quadrant). When the effects had opposite signs, confounding bias was negative. The magnitude of confounding bias increased as the confounder-exposure or confounder-outcome effect increased in magnitude. There was no confounding bias when one or both effects equaled zero.

### The noncollapsibility effect

**Fig. 3:**
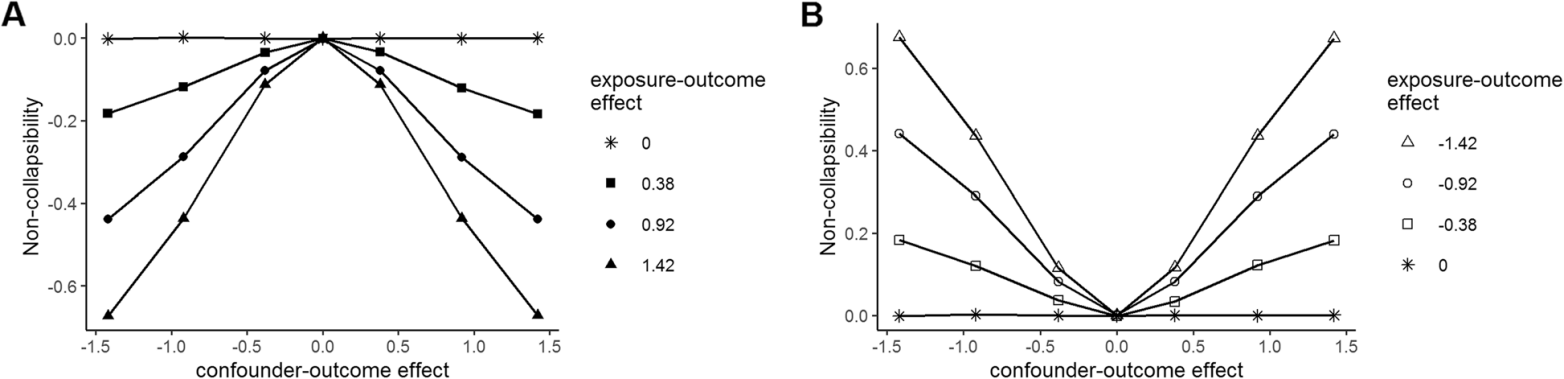
The noncollapsibility effect (${{\varvec{\beta}}}_{1}^{\boldsymbol{*}}-{{\varvec{\beta}}}_{1}^{\boldsymbol{^{\prime}}}$) as a function of the confounder-outcome effect collapsed over all sample sizes. Panel **A**: each line represents a positive exposure-outcome effect. Panel **B**: each line represents a negative exposure-outcome effect

Figure 3 plots the noncollapsibility effect (${\beta }_{1}^{*}-{\beta }_{1}^{^{\prime}}$) as a function of the confounder-outcome effect with the lines in panel A representing positive exposure-outcome effects of various magnitudes and the lines in panel B representing negative exposure-outcome effects of various magnitudes. The noncollapsibility effect and the exposure-outcome effect were inversely related: when the latter effect was positive, the noncollapsibility effect was negative, and vice versa. The noncollapsibility effect increased in magnitude as both the exposure-outcome effect and the confounder-outcome effect increased in magnitude. When either effect was zero, there was no noncollapsibility effect, regardless of the magnitude of the other effect.

## Empirical data example

**Fig. 4:**
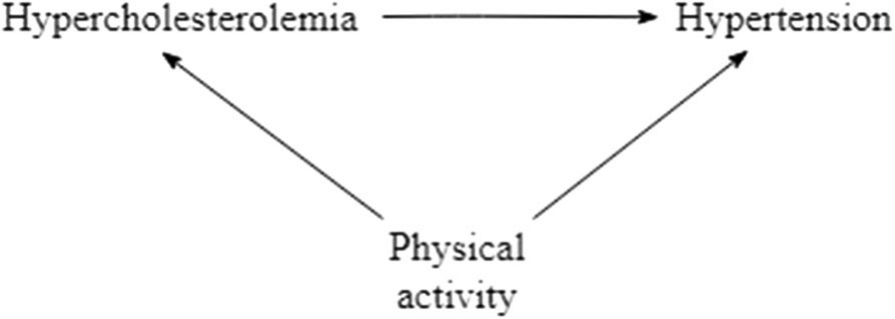
The assumed relations between hypercholesterolemia, hypertension and physical activity

To illustrate how the noncollapsibility effect might affect conclusions about confounding bias in practice we use an example from the Amsterdam Growth and Health Longitudinal Study (AGHLS). The AGHLS started in 1976 with the aim to examine growth and health among teenagers. Over the years, health and lifestyle measures, determinants of chronic diseases and parameters for the investigation of deterioration in health with age have been measured [33]. The data in this example were collected in 2000, when the participants were in their late 30s. Using data from the AGHLS we investigated the association between hypercholesterolemia and hypertension, potentially confounded by physical activity. Using multivariable regression analysis and IPW we estimated the confounder-adjusted effect of hypercholesterolemia on hypertension in our sample, ${\beta }_{1}^{^{\prime}}$ and ${\beta }_{1}^{*}$, respectively. To quantify the magnitude of confounding bias and the noncollapsibility effect, we also estimated the unadjusted exposure effect ${\beta }_{1}$ using univariable regression analysis. Cut-offs for hypercholesterolemia and hypertension were based on guidelines from the U.S. National Institutes of Health (NIH) and NIH’s National Heart, Lung and Blood Institute, respectively [34, 35]. Physical activity was defined as the total hours per week spent on light, moderate or vigorous activities. Only subjects with complete data on the variables were considered in the analysis (n = 349). Figure 4 provides a graphical representation of the assumed relations among the variables.

Table 2 shows the effect estimates from univariable- and multivariable regression analysis and IPW. The unadjusted effect estimate ${\beta }_{1}$ was 0.90, corresponding to an odds ratio (OR) of 2.46. The multivariable confounder-adjusted exposure effect estimate ${\beta }_{1}^{^{\prime}}$ was 0.93, corresponding to an OR of 2.53. The IPW confounder-adjusted exposure effect estimate ${\beta }_{1}^{*}$ was 0.99, corresponding to an OR of 2.69.

**Table 2:** Relationship between hypercholesterolemia and hypertension estimated using univariable- and multivariable regression analysis and IPW

	$\beta$	$SE(\beta )$	$95\% CI$	$P$
Univariable exposure effect				
Hypercholesterolemia	0.90	0.23	0.47; 1.35	< 0.01
Multivariable confounder-adjusted exposure effect				
Hypercholesterolemia	0.93	0.23	0.48; 1.38	< 0.01
Physical activity	0.01	0.01	-0.02; 0.03	0.60
IPW confounder-adjusted exposure effect				
Hypercholesterolemia	0.99	0.16	0.69; 1.30	< 0.01

Abbreviations: SE: standard error; CI: confidence interval

The difference between ${\beta }_{1}^{^{\prime}}$ and ${\beta }_{1}$ was -0.03, or 3.3%. If one would use the change-in-estimate criterion with a cut-off of 10% to determine the presence of confounding, then physical activity would not be considered a confounder. Using the difference between ${\beta }_{1}$ and ${\beta }_{1}^{*}$, the estimate of confounding bias was 0.90 – 0.99 = -0.09. This corresponds to a 10% change in the exposure effect estimate. The noncollapsibility effect estimate was 0.99 – 0.93 = 0.06. Because of this noncollapsibility effect, the estimate of the true confounding bias of physical activity was considerably larger than it seemed based on the difference between ${\beta }_{1}^{^{\prime}}$ and ${\beta }_{1}$. Thus, in our data example, the conventional method to determine the presence of confounding led to an underestimation of the true confounding bias of physical activity.

## Discussion

This paper aimed to clarify the role of noncollapsibility in determining the magnitude of confounding bias in logistic regression. Because the difference between ${\beta }_{1}^{^{\prime}}$ and ${\beta }_{1}$ reflects both confounding bias and a noncollapsibility effect, in logistic regression the change-in-estimate criterion should not be used to determine the presence of confounding. This was illustrated in our data example, in which confounding bias was underestimated because of the magnitude of the noncollapsibility effect. Our simulation study showed that confounding was mainly determined by the combination of the magnitude of the confounder-exposure and confounder-outcome effects, whereas noncollapsibility was mostly determined by the magnitude of the combination of the exposure-outcome and confounder-outcome effects. In situations in which confounding approached zero and noncollapsibility was non-zero, the change-in-estimate criterion wrongly indicated the presence of confounding bias, when in reality the difference between ${\beta }_{1}^{^{\prime}}$ and ${\beta }_{1}$ was caused solely by the noncollapsibility effect.

### Recommendations for practice

Rather than using an arbitrary statistical rule such as the 10% cut-off based on the change-in-estimate criterion, it is generally recommended to determine the confounder set based on subject matter knowledge. Directed acyclic graphs (DAGs) are helpful to determine which set of confounders should be adjusted for to eliminate confounding bias [36, 37]. DAGs are causal diagrams in which the arrows represent the causal relations among variables. Therefore, DAGs contain information about the causal model that cannot be provided by statistical methods. For example, assuming the DAG is a correct representation of the causal relations among variables, it clarifies what the minimally sufficient set of confounders is to block any backdoor paths (i.e., confounding paths) from the exposure to the outcome. The amount of confounding bias could be quantified by looking at the difference between the unadjusted univariable exposure effect estimate ${\beta }_{1}$ and the IPW confounder-adjusted exposure effect estimate ${\beta }_{1}^{*}$ as proposed by Pang et al. [7] and Janes et al. [9]. Bootstrap confidence intervals can be used to determine the statistical significance of the confounding bias.

Because of the noncollapsibility effect, multivariable regression analysis and IPW provide different estimates of the exposure effect. Multivariable regression analysis results in a *conditional* exposure effect estimate [16, 38], whereas IPW results in a *population-average* or *marginal* exposure effect estimate [16, 38–40]. Marginal exposure effects can also be estimated with standardization using G-computation. A step-by-step demonstration of G-computation can be found elsewhere [41]. It is often suggested that a population-average effect estimate should be reported when the target population is the entire study population, while the conditional exposure effect should be reported if the target population is a subset of the study population [7, 8, 16, 38, 39, 42, 43]. Although this distinction is known from the literature, when it comes to the practical application, the exact differences between the two exposure effect estimates and their respective interpretations remain unclear.

In this study, we assume correct specification of both the confounder-exposure and the confounder-outcome effect. When these are not correctly specified, bias might be introduced and the difference between the unadjusted univariable exposure effect estimate and the IPW confounder-adjusted exposure effect estimate might not only reflect confounding bias but also the misspecification of the underlying models. Therefore, correct specification of all effects is necessary to estimate unbiased exposure effects and correctly quantify confounding bias.

## Conclusion

To summarize, in this study we showed that in logistic regression the difference between univariable- and multivariable effect estimates may reflect both confounding bias and a noncollapsibility effect. To avoid wrong conclusions with respect to the magnitude and presence of confounding bias, confounders are ideally determined based on subject matter knowledge. To quantify confounding bias, one could look at the difference between the unadjusted univariable exposure effect estimate and the IPW confounder-adjusted exposure effect estimate.

## Supplementary Information

**Additional file 1.** Hypothetical data example to illustrate the noncollapsibility effect.

**Additional file 2.** Code used to generate data for the simulation study.

## Data Availability

1. The dataset used and analyzed in the empirical data example is available from the corresponding author on reasonable request. 2. The code for the simulated dataset is included in this study in additional file B. 3. The detailed results of the simulation study are available on request from the corresponding author.
