# Supplementary material for: Noncollapsibility and its role in quantifying confounding bias in logistic regression
Source: BMC Med Res Methodol. 2021 Jul 5;21:136. doi: 10.1186/s12874-021-01316-8 (PMC8259440; doi:10.1186/s12874-021-01316-8)
Supplement: Supplementary file 1 — Additional file 1. Hypothetical data example to illustrate the noncollapsibility effect. [file 12874_2021_1316_MOESM1_ESM.docx]

**Additional file A Hypothetical example**

This hypothetical example involves three dichotomous variables: the exposure variable weight (not overweight vs. overweight), the event of interest diabetes and the potential confounding variable sex. The total population consists of 200 individuals. The data can be summarized according to Table A.1.

Of these 200 individuals, half are overweight and half are not overweight. A total of 80 individuals have diabetes and 120 individuals do not have diabetes. The unadjusted exposure effect, estimated with univariable regression analysis, is 1.299. This corresponds to an odds ratio of 3.667.

The sexes are evenly distributed over the weight groups: both groups consist of 50 males and 50 females. Because there are equal numbers of males and females in the overweight and not overweight group, weight status is not influenced by sex. In contrast to sex and weight, sex and diabetes are associated. Of the 100 women, 30 have diabetes and 70 do not, whereas for males half have diabetes and half do not. Because confounding requires the covariate to be associated with both the exposure and the outcome, sex is not a confounder in the relation between weight and diabetes, as it is not associated with weight.

Since sex is not a confounder of the exposure-outcome effect, adjustment for sex should not affect the exposure-outcome effect estimate. However, the adjusted exposure effect estimate, estimated with multivariable regression analysis, is 1.366, corresponding to an odds ratio of 3.921. This is different from the unadjusted effect estimate of 1.299. The results from all analyses are shown in Table A.2.

Although sex is not a confounder, the effect estimates from univariable- and multivariable regression analysis still differ. This difference of 0.067 is entirely caused by noncollapsibility. This example illustrates that, even in the absence of confounding, the univariable and multivariable exposure effect estimates might differ. Therefore, the change-in-estimate based on logistic regression coefficients may lead to wrong conclusions when used to determine the presence of confounding.

**Table A.1** Hypothetical data example

| Exposure | Event | Sex | *n* |
| --- | --- | --- | --- |
| Not overweight | No diabetes | Female | 45 |
| Not overweight | Diabetes | Female | 5 |
| Not overweight | No diabetes | Male | 30 |
| Not overweight | Diabetes | Male | 20 |
| Overweight | No diabetes | Female | 25 |
| Overweight | Diabetes | Female | 25 |
| Overweight | No diabetes | Male | 20 |
| Overweight | Diabetes | Male | 30 |

Abbreviations: n: sample size

**Table A.2** Association between sex and weight, sex and diabetes and weight and diabetes

|  | $\beta$ | *OR* | *95% CI* |
| --- | --- | --- | --- |
| *Association between sex and weight* | | | |
| Sex | < 0.001 | 1 | 0.574; 1.742 |
|  |  |  |  |
| *Association between sex and diabetes* | | | |
| Sex | 0.847 | 2.333 | 1.313; 4.201 |
|  |  |  |  |
| *Association between weight and diabetes* | | | |
| Univariable exposure effect | 1.299 | 3.667 | 2.031; 6.765 |
| Multivariable exposure effect | 1.366 | 3.921 | 2.133; 7.402 |

Abbreviations: OR: odds ratio; CI: confidence interval (around OR)
