# Supplementary material for: Noncollapsibility and its role in quantifying confounding bias in logistic regression
Source: BMC Med Res Methodol. 2021 Jul 5;21:136. doi: 10.1186/s12874-021-01316-8 (PMC8259440; doi:10.1186/s12874-021-01316-8)
Supplement: Supplementary file 2 — Additional file 2. Code used to generate data for the simulation study. [file 12874_2021_1316_MOESM2_ESM.docx]

**Additional file B Simulation code**

**Step 1 – generate data**

generate_data <- function(seed, reps, n, ix, iy, cx, xy, cy){

# define total number of rows required to store data

rows <- n * reps

# create data frame to store data in

df <- as.data.frame(matrix(NA, nrow = rows, ncol = 14))

colnames(df) <- c("ID", # ID through entire data set

"repnr", # for each repetition

"ID_repnr", # ID through each repetition

"n", # number of observations

"ix", # intercept exposure

"iy", # intercept outcome

"cx", # CX effect

"xy", # XY effect

"cy", # CY effect

"C1", # confounder 1

"C2", # confounder 2

"C3", # confounder 3

"X", # dichotomous exposure

"Y") # dichotomous outcome

# define simulation parameters

df[, "ID"] <- seq(1:rows)

df[, "repnr"] <- rep(1:reps, each = n)

df[, "ID_repnr"] <- rep(seq(1, n), reps)

df[, "n"] <- n

# define intercepts ix and iy

df[, "ix"] <- ix

df[, "iy"] <- iy

# define coefficients a, b and c

df[, "cx"] <- cx

df[, "xy"] <- xy

df[, "cy"] <- cy

# generate confounders C1, C2 and C3

df[, "C1"] <- rnorm(n = rows)

df[, "C2"] <- rnorm(n = rows)

df[, "C3"] <- rnorm(n = rows)

# generate dichotomous exposure X

lpx <- ix + cx * df[, "C1"] + cx * df[, "C2"] + cx * df[, "C3"]

prx <- 1/(1 + exp(-lpx))

df[, "X"] <- rbinom(n = rows, size = 1, prob = prx)

# generate dichotomous outcome Y

lpy <- iy + xy * df[, "X"] + cy * df[, "C1"] + cy * df[, "C2"] + cy * df[, "C3"]

pry <- 1/(1 + exp(-lpy))

df[, "Y"] <- rbinom(n = rows, size = 1, prob = pry)

# transform X and Y into factor variables

df[, "X"] <- factor(df[, "X"])

df[, "Y"] <- factor(df[, "Y"])

# return data frame

return(df)

}

# define simulation parameters

seed <- 20200908

reps <- 1000

n <- c(250, 500, 750, 1000)

ix <- 0

iy <- 0

cx <- c(-1.42, -0.92, -0.38, 0, 0.38, 0.92, 1.42)

xy <- c(-1.42, -0.92, -0.38, 0, 0.38, 0.92, 1.42)

cy <- c(-1.42, -0.92, -0.38, 0, 0.38, 0.92, 1.42)

for(i in n){

for(j in cx){

for(k in xy){

for(l in cy){

df <- generate_data(seed = seed,

reps = reps,

n = i,

ix = ix,

iy = iy,

cx = j,

xy = k,

cy = l)

# save each file in folder 'Step 1 - Generated datasets'

save(df, file = paste0("Step 1 - Generated datasets\\",

"n = ", i,", cx = ", j, ", xy = ", k, ", cy = ", l, ".RData"))

}

}

}

}

**Step 2 – generate models**

library(dplyr)

generate_models <- function(data){

# create data frame to store effect estimates in

effects <- data.frame(matrix(NA, nrow = max(data$repnr), ncol = 8))

colnames(effects) <- c("repnr",

"n",

"cx",

"xy",

"cy",

"coef_univar",

"coef_multivar",

"coef_ipw")

# store simulation characteristics

effects$repnr <- unique(data$repnr)

effects$n <- unique(data$n)

effects$cx <- unique(data$cx)

effects$xy <- unique(data$xy)

effects$cy <- unique(data$cy)

# FIT MODELS

# 1. univariable regression model

estimates <- data %>%

group_by(repnr) %>%

do(model_univar = glm(Y ~ X, family = "binomial", data = .)$coefficients[2])

effects$coef_univar <- unlist(estimates$model_univar)

# 2. multivariable regression model

estimates <- data %>%

group_by(repnr) %>%

do(model_multivar = glm(Y ~ X + C1 + C2 + C3, family = "binomial", data =

.)$coefficients[2])

effects$coef_multivar <- unlist(estimates$model_multivar)

# 3. inverse probability weighting

estimates <- data %>%

group_by(repnr) %>%

do(ps = predict(glm(X ~ C1 + C2 + C3, family = "binomial", data = .), type =

"response"))

data$ps <- unlist(estimates$ps)

data$weights <- ifelse(data$X == 1, 1/data$ps, 1/(1 - data$ps))

data$stab_weights <- data$weights/sum(data$weights)

estimates <- data %>%

group_by(repnr) %>%

do(model_ipw = glm(Y ~ X, weights = stab_weights, family = "binomial", data =

.)$coefficients[2])

effects$coef_ipw <- unlist(estimates$model_ipw)

# return data frame with simulation characteristics and treatment effects

return(effects)

}

# save path

path <- "Step 1 - Generated datasets\\"

# save all file names in files

files <- list.files(path = path, pattern = "*.RData")

# loop through each file in the folder

for(i in files){

# load the data into the environment

load(paste0(path, i))

# run function

effects <- generate_models(df)

# save each file in folder 'Step 2 - Generated models'

save(effects, file = paste0("Step 2 - Generated models\\", i))

}

**Step 3 – merge data**

# save path

path <- "Step 2 - Generated models\\"

# save all file names in files

files <- list.files(path = path, pattern = "*.RData")

# load first dataset (effects) of files

load(paste0(path, files[1]))

# rename dataset (effects) to df

df <- effects

rm(effects)

# append all other files to current file df

for(i in paste0(path, files[-1])){

load(i)

df <- rbind(df, effects)

rm(effects)

}

# add scenario number for each scenario (total = 1372)

df$scenario <- rep(seq(from = 1, to = nrow(df)/1000), each = 1000)

# change column order

df <- df[c("scenario", "repnr", "n", "cx", "xy", "cy", "coef_univar", "coef_multivar", "coef_ipw")]

# save appended file df in folder 'Step 3 - Appended file'

save(df, file = "Step 3 - Appended file\\Appended file (all scenarios - all effect measures).RData")

**Step 4 – confounding decomposition**

# df contains the treatment effects derived from the 3 methods, calculated for each repetition (n = 1000) within each scenario (n = 1372)

load("Step 3 - Appended file\\Appended file (all scenarios - all effect measures).RData")

# calculate the difference between the unadjusted exposure effect and the conditional exposure effect

df$diff <- df$coef_univar - df$coef_multivar

# calculate the true confounding effect

df$true_conf <- df$coef_univar - df$coef_ipw

# calculate the amount of non-collapsibility

df$non_collaps <- df$coef_ipw - df$coef_multivar

# save file containing the confounding decomposition in folder 'Step 4 - Confounding decomposition'

save(df, file = "Step 4 - Confounding decomposition\\Final dataset.RData")
